# Acetabular cup‐native femoral head size discrepancy in primary total hip arthroplasty: A meta‐analysis on intraoperative sizing verification

**DOI:** 10.1002/jeo2.70782

**Published:** 2026-05-22

**Authors:** Chahine Assi, Ralph Maroun, Kaissar Yammine, Pascal Kouyoumdjian, Youssef Jamaleddine

**Affiliations:** ^1^ Department of Orthopedic Surgery Lebanese American University Medical Center‐Rizk Hospital Beirut Lebanon; ^2^ Department of Orthopedic Surgery Lebanese University Beirut Lebanon; ^3^ Orthopedic and Trauma Surgery Department Hospital and University Center of Caremeau Nîmes, Rue du Professeur Robert Debré Nîmes France; ^4^ Laboratory of Mechanics and Civil Engineering (LMGC), CNRS‐UM1 Montpellier France

**Keywords:** acetabular cup sizing, femoral head diameter, intraoperative measurement, templating, total hip arthroplasty

## Abstract

**Purpose:**

Intraoperative measurement of the native femoral head has been proposed as a simple cross‐check for acetabular cup sizing in total hip arthroplasty (THA), but reported cup‐head discrepancies vary. We synthesized the evidence to define an expected range and a pragmatic intraoperative alert threshold.

**Methods:**

A Preferred Reporting Items for Systematic Reviews and Meta‐Analyses (PRISMA)‐guided search of Cochrane Library, MEDLINE, Embase, Web of Science and Google Scholar (pages 1–20) from inception to 24 December 2025 identified studies reporting intraoperative femoral head diameter and implanted acetabular cup size. The primary outcome was the mean difference (Δ) between cup size and head diameter. Data were pooled using a random‐effects model, 95% confidence interval (CI) and both 95% and 75% prediction interval (PI) were calculated.

**Results:**

Four studies (631 hips; mean age 66 years) were included. The pooled mean Δ was 2.93 mm (standard error [SE] 0.46). The 95% CI was 2.03–3.83 mm, and the 95% PI was −1.45 to 7.32 mm, indicating between‐study variability. The 75% PI ranged from 1.30 to 4.57 mm. Across 556 hips with categorical data, the cup exceeded the head in 87.77% of cases; Δ was 0–2 mm in 36.33%, 2–4 mm in 33.27%, exactly 0 mm in 9.35%, >4 mm in 18.17% and negative in 2.88%.

**Conclusion:**

In primary THA, acetabular cups are around 2.93 mm larger than the excised femoral head, most often between 0 and 4 mm. This relationship supports femoral‐head sizing as a rapid, no‐cost intraoperative cross‐check of reaming accuracy and final cup selection. A cup‐head mismatch >4 mm should trigger verification of measurement, reaming progression and templating assumptions before altering component size.

**Level of Evidence:**

Level IV, systematic review and meta‐analysis.

**Abbreviations**
Δ: mean difference (Δ = cup size − head diameter)
2D: two‐dimensional
3D: three‐dimensional
CI: confidence interval
CMA: Comprehensive Meta‐Analysis (software)
COR: centre of rotation
CT: computed tomography
JBI: Joanna Briggs Institute
PI: prediction interval
PRISMA: Preferred Reporting Items for Systematic Reviews and Meta‐Analyses
SE: standard error
THA: total hip arthroplasty

## INTRODUCTION

Total hip arthroplasty (THA) is one of the most effective surgical operations for restoring functional mobility to a wide range of musculoskeletal conditions [30]. The fundamental principle of this surgery is to re‐establish the joint's native biomechanics by reinstituting the centre of rotation (COR), ensuring prosthesis longevity [14, 16]. Despite its recognized success, THA faces a variety of technical challenges, such as improper component size selection or anatomical positioning [8, 28]. These surgical difficulties are directly linked to mechanical complications such as peri‐prosthetic fractures, instability and leg length discrepancies [4, 6, 29]. Therefore, precise preoperative planning is essential.

To minimize the risk of these adverse outcomes, surgeons employ various methods that have been developed to ensure the correct size of the implanted acetabular cup [11]. For example, acetabular component oversizing may produce anterior cup rim overhang, which has been associated with postoperative groin pain due to iliopsoas tendinopathy [19]. Similarly, acetabular oversizing can increase posterior rim overhang, which may contribute to posterior impingement [18]. Acetabular oversizing may alter the hip COR, as reported with jumbo‐cup implantation [22]. Traditionally, cup size prediction involved using acetate templates on standard printed film radiographs [13]. With the emergence of new technologies, digital templating was adopted using sophisticated software provided by various manufacturing companies, capable of two‐dimensional (2D) or three‐dimensional (3D)‐based planning of cup sizes [2, 27]. More recently, semiautomated segmentation workflows using open‐source software have enabled patient‐specific 3D‐printed pelvic and acetabular models for preoperative planning and education in primary and revision hip arthroplasty [24]. All of these approaches are inherently pre‐operative techniques, relying either on radiographic imaging with associated magnification errors and variability in patient positioning or on costly software platforms that may not be readily available and require additional resources and training [10, 13, 31]. Accordingly, intraoperative measurement of the excised femoral head has been described as an additional real‐time intraoperative monitoring tool to support acetabular cup size selection alongside preoperative planning [3]. This method has been proposed as a cost‐free, simple intraoperative reference to support acetabular cup‐size selection independent of radiographic limitations and technical constraints [1].

Despite the benefits of intra‐operative measurements, the current research shows variable results regarding the ideal difference between the implanted acetabular cup and the native femoral head diameter. For example, Assi et al. found a mean difference of 1.9 mm and recommended a safety limit of 2.5 mm to prevent oversizing [1]. In contrast, Ben Lulu et al., Kouyoumdjian et al. and Mahamud et al. found larger differences, averaging between 3.2, 3.14 and 3.52 mm, respectively, with Ben Lulu and Mahamud proposing a more permissive monitoring threshold of 4 mm [3, 12, 21]. Additionally, while some researchers believe that manual head sizing is as reliable as digital templating for preventing oversized implants, others argue it lacks the precision of computed tomography (CT)‐based templating [12]. Many of these studies are limited by small sample sizes. A meta‐analysis is needed to combine these varied findings into a single, evidence‐based guideline for surgeons.

## MATERIAL AND METHODS

### Search strategy

**Figure 1:**
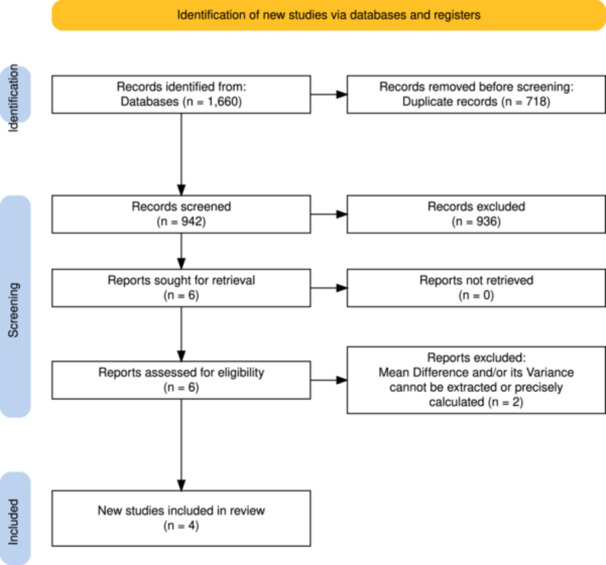
PRISMA flow diagram of study selection. PRISMA, Preferred Reporting Items for Systematic Reviews and Meta‐Analyses.

This meta‐analysis was performed in accordance with the Preferred Reporting Items for Systematic Reviews and Meta‐Analyses (PRISMA) guidelines [23]. A comprehensive literature search was conducted using the Cochrane Library, MEDLINE, Embase, Web of Science and Google Scholar (pages 1–20), covering all publications from the database's inception through 24 December 2025. Search terms related to native femoral head measurement for acetabular cup size selection were combined using Boolean operators (‘AND’ and ‘OR’), including ‘femoral head’, ‘measurement’, ‘acetabular cup’, ‘cup size’, ‘acetabular component’, ‘hip replacement’ and ‘hip arthroplasty’. Additionally, reference lists of the included studies were manually screened to identify any further relevant articles. The study selection process is illustrated in the PRISMA flow diagram (Figure 1).

### Eligibility criteria

Studies were eligible for inclusion if they were published in English or non‐English languages and reported measurements of the native femoral head diameter along with the corresponding implanted acetabular cup size in patients undergoing hip arthroplasty. Non‐original research articles, including case reports, review articles and editorials, were excluded. Studies not directly reporting the mean difference between implanted acetabular cup size and native femoral head diameter were excluded if they did not report sufficient quantitative data to calculate the mean difference. Therefore, the PICO framework on which this study was structured is as follows: Population = adult patients undergoing primary THA; Index measurement = intraoperative native femoral head diameter; Comparator = final implanted acetabular cup size; Outcome = mean difference (Δ, in mm) between implanted cup size and intraoperative femoral head diameter.

Study selection was performed independently by two reviewers. Any disagreements regarding eligibility were resolved through discussion, with arbitration by a third senior reviewer when consensus could not be reached.

### Data extraction

Data extraction and tabulation were performed using a Microsoft Excel spreadsheet (Version 2021; Microsoft).

Extracted data focused on the quantitative difference between the implanted acetabular cup size and the intraoperative measurement of the native femoral head diameter, including the mean difference and corresponding standard deviation.

Extracted study characteristics included patient demographics and the mean duration of final follow‐up. Surgery‐related characteristics comprised the implant manufacturer and model, acetabular cup type and material, surgical approach, any reported recommendations for acetabular cup oversizing thresholds and the number of operating surgeons. Additionally, when available, the number of implanted acetabular cups corresponding to each specific Δ value of interest was also extracted or calculated from provided raw data after contacting the corresponding author; Δ being the quantitative difference between the implanted acetabular cup size and the intraoperative measurement of the native femoral head diameter.

### Risk of bias assessment

Risk of bias was assessed using the Joanna Briggs Institute (JBI) Critical Appraisal Checklist for Analytical Cross‐Sectional Studies [20]. Two reviewers independently evaluated each included study across eight specific items, focusing on the clarity of inclusion criteria, the validity and reliability of exposure and outcome measurements, the identification and management of confounding factors and the appropriateness of the statistical analysis. Any disagreements were resolved through consensus or consultation with a senior reviewer.

### Statistical analysis

The statistical analysis was conducted using Comprehensive Meta‐Analysis Version 4 (CMA v4) software to synthesize data across the included studies [5]. The primary outcome of interest was the pooled mean difference, defined as the final implanted acetabular cup size minus the intra‐operative native femoral head diameter, measured in millimetres. To calculate this, the mean difference and its corresponding standard error (SE) were extracted or calculated from each study and pooled using generic inverse variance statistics.

A random‐effects model was utilized for the analysis to account for expected heterogeneity between studies, arising from variations in surgical assessments and patient demographics. This model provided a more conservative estimate of the true effect size by incorporating both within‐study and between‐study variance. Heterogeneity was estimated using the DerSimonian–Laird (DL) method, as implemented in CMA v4, and quantified using the *Q* statistic, *I* ^2^ statistic, *τ* ^2^ and *τ*. Publication bias was not formally assessed via funnel plot or Egger's test, as the number of included studies (*n* = 4) falls below the minimum of 10 studies conventionally required for these methods to yield reliable results.

The results of the meta‐analysis were visualized through forest plots, reporting the pooled mean difference alongside multiple statistical intervals. For better scientific validation, 95% confidence intervals (CIs) and 95% prediction intervals (PIs) were calculated. Additionally, to enhance clinical utility and provide surgeons with more practical thresholds for intra‐operative decision‐making, 75% CI and 75% PI were also reported. While the CI indicates the precision of the mean estimate, the PI is particularly critical in this context as it predicts the range in which the difference between the implanted acetabular cup size and the intraoperative measurement of the native femoral head diameter for a future population of THA patients is likely to fall.

## RESULTS

### Characteristics of the included studies

Four studies met the inclusion criteria [1, 3, 12, 21]. These studies included 631 hips with an overall mean age of 66 years. The main characteristics of the included studies are summarized in Table 1. The results of the risk‐of‐bias assessment of the included studies are summarized in Table 2.

**Table 1:** Main characteristics of included studies.

Paper	Number of patients	Mean age (years)	Latest follow‐up period (months)	Implant brand(s) and model	Cup type/material	Surgical approach	Reaming technique	Suggested ‘oversize’ cut‐off	Number of surgeons
Assi et al. (2024)	154 patients (157 hips)	66.2 ± 10.4	19.5 (range: 1–48)	Groupe Lepine: Quattro PnP SpikeFit	Dual Mobility; Cementless hemispherical; Bilayer coating (porous titanium and hydroxyapatite)	Posterolateral	Line‐to‐line technique	>2.5 mm	One
Ben Lulu et al. (2015)	75 patients (75 hips)	64.3 ± 11.2	19.5 (range: 1–48)	Pinnacle (34), Trinity (32), Allofite (7), Exceed (2)	Cementless; 67% Ceramic liners; 33% Polyethylene; 88% without screws	Direct Lateral (Hardinge)	N/A	>4 mm	Two
Kouyoumdjian et al. (2025)	619 patients (299 hips)	67.6 ± 12.2	N/A	Stryker: Trident‐II or Tritanium	Cementless hemispherical	N/A	Single reamer (diameter matches final cup size)	N/A	Two
Muñoz‐Mahamud et al. (2022)	100 patients (100 hips)	65.1 ± 13.3	N/A	Smith & Nephew R3 (45), Zimmer Biomet G7 (39), Stryker Trident (13), Stryker X3 RimFit (2)	98% Cementless; 2% cemented; Mixed liner types	Anterolateral (70), Direct Anterior (18), Posterolateral (12)	Progressive acetabular reaming	>4 mm	Unspecified

Abbreviation: N/A, not applicable.

**Table 2:** Risk‐of‐bias assessment of the included studies.

Checklist question	Assi et al. (2024)	Ben Lulu et al. (2015)	Kouyoumdjian et al. (2025)	Muñoz‐Mahamud et al. (2022)
1. Were inclusion criteria clearly defined?	Yes	Yes	Yes	Yes
2. Were study subjects and setting described?	Yes	Yes	Yes	Yes
3. Was exposure measured validly/reliably?	Yes	Yes	Yes	Yes
4. Were objective, standard criteria used?	Yes	Yes	Yes	Yes
5. Were confounding factors identified?	Yes	Yes	Yes	No
6. Were strategies to deal with them stated?	Yes	Yes	Yes	Unclear
7. Were outcomes measured validly/reliably?	Yes	Yes	Yes	Yes
8. Was appropriate statistical analysis used?	Yes	Yes	Yes	Yes
Total number of ‘Yes’	8/8	8/8	8/8	6/8

### Meta‐analysis of sizing discrepancies (forest plots)

**Figure 2:**
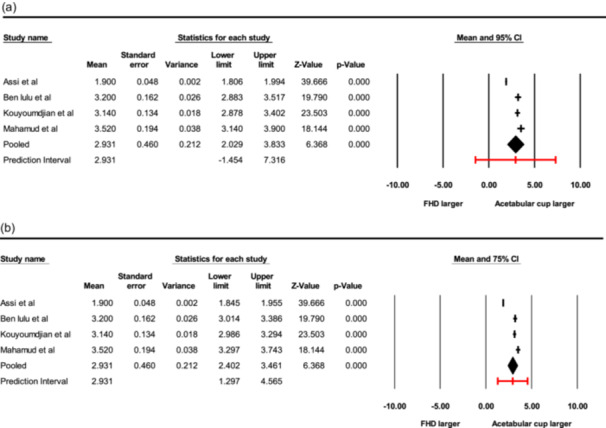
(a) Forest plot of pooled mean difference (Δ) with 95% CI and prediction interval. (b) Forest plot of pooled mean difference (Δ) with 75% CI and prediction interval. CI, confidence interval; FHD, femoral head diameter.

The meta‐analysis of the four included studies, involving a total of 631 hips, yielded a pooled mean difference (Δ) of 2.93 mm (SE: 0.46, *Q* = 173.23, df = 3, *p* < 0.001; *I* ^2 ^= 98%; *τ* ^2^ = 0.827; *τ* = 0.909) (Figure 2a). This result confirms that the implanted acetabular cup is significantly larger than the intraoperatively measured native femoral head.

- 95% Statistical validation: Using a random‐effects model, the 95% CI for the pooled mean difference was 2.03 to 3.83 mm. The 95% PI, which estimates the range of the sizing difference for a future studied population of patients, was broader, ranging from −1.45 to 7.32 mm (Figure 2a).
- 75% Clinical utility: To provide surgeons with a more practical intra‐operative guideline, intervals were also calculated at the 75% level. The 75% CI was 2.40 to 3.46 mm, while the 75% PI resulted in a range of 1.30–4.57 mm. This tighter PI suggests that in the majority (i.e., 75%) of any future population of THA studied, the ‘delta’ will likely fall between approximately 1.3 and 4.6 mm (Figure 2b).

### Categorical distribution of sizing discrepancies

The distribution of the difference (Δ) between the final implanted cup and the native femoral head was analysed across a combined cohort of 556 hips from three of the included studies [1, 12, 21]. The aggregated data showed that positive discrepancies (implanted cup > native femoral head) were dominant, occurring in 87.77% of all cases (Table 3).

- Primary distribution: The most frequent sizing outcome was an implanted cup larger than the native femoral head by a difference >0 but ≤2 mm, seen in 36.33% of patients, followed closely by a discrepancy that is >2 but ≤4 mm, at 33.27%.
- Exact matches: A perfect match between the intra‐operatively measured native femoral head and the final implanted cup (*Δ* = 0) was achieved in 9.35% of the population.
- The ‘Caution Zone’: High‐magnitude discrepancies where the implanted cup was more than 4 mm larger than the native femoral head were recorded in 18.17% of the total cohort.
- Under‐sizing: Instances where the implanted cup was smaller than the native femoral head (negative Δ) were rare, accounting for only 2.88% of the total surgeries.

**Table 3:** Categorical distribution of the difference (Δ) in mm between final implanted acetabular cup size and intra‐operative native femoral head diameter.

Difference (Δ) range^a^	Assi et al. (*n* = 157)	Muñoz‐Mahamud (*n* = 100)	Kouyoumdjian (*n* = 299)	Total (*N* = 556)	Combined %
Δ < −4	0	0	0	0	0.00%
−4 ≤ Δ < −2	0	0	2	2	0.36%
−2 ≤ Δ < 0	8	3	3	14	2.52%
Δ = 0 (exact match)	22	2	28	52	9.35%
0 < Δ ≤ 2	86	26	90	202	36.33%
2 < Δ ≤ 4	34	36	115	185	33.27%
Δ > 4	7	33	61	101	18.17%
Total	157	100	299	556	100.00%

^a^ For Ben Lulu et al., Δ distribution not reported: data unavailable and no categorical breakdown provided.

## DISCUSSION

Restoring native hip biomechanics is a essential for durable function after THA, particularly through accurate reconstruction of the acetabular COR, leg length and femoral offset [14, 15]. COR and offset restoration influence joint reaction forces, abductor tension, lever arm, gait mechanics and stability, which in turn affects wear, loosening, gait and functional outcomes [14, 15, 26]. Contemporary technique combines preoperative templating to anticipate implant needs, and intraoperative checking to detect when anatomy, imaging or execution diverge from plan [7, 14].

In this meta‐analysis, pooling 631 hips, the pooled mean difference, defined as the implanted acetabular cup size minus the intraoperatively measured native femoral head diameter, was 2.93 mm [1, 3, 12, 21]. The 95% PI was broad (−1.45 to 7.32 mm), indicating clinically meaningful between‐study variation; whereas the more practice‐oriented 75% PI suggested that in most future series the discrepancy in size will likely fall between 1.3 and 4.57 mm. This supports that the femoral‐head‐based intraoperative sizing behaves less like a target number and more like an intraoperative real‐time plausibility check that flags outliers relative to the expected practice ranges.

Included studies explicitly reflected two practical warning thresholds. Assi et al. recommended caution beyond roughly 2.5 mm [1]. Notably, this was a single‐surgeon study using a same surgical approach and a same acetabular cup design. While Muñoz‐Mahamud et al. concluded that ‘utmost caution’ is warranted when the reamer exceeds the native head by >4 mm, aligning with the monitoring threshold suggested by Ben lulu et al. [3, 21]. Nonetheless, these studies involved multiple surgeons and/or different acetabular cup designs. Analysis of the 556 hips pooled from three of the included studies suggests that sizing discrepancies are not concentrated around a single ideal size difference [1, 12, 21]. Instead, the distribution reveals two dominant and nearly equal bands, 0 < Δ ≤ 2 mm (36.33%) and 2 < Δ ≤4 mm (33.27%). Together, these account for 69.6% of cases, roughly two‐thirds, indicating that, in routine primary THA, the most common scenario is an implanted cup that is modestly larger than the excised femoral head by 0–4 mm. In contrast, exact matches (Δ = 0) were relatively uncommon (9.35%), while negative size differences, where the cup is smaller than the head, were rare (2.88%). However, this may be encountered in hips with a ‘mushroom’ femoral head, in which peripheral rim hypertrophy enlarges the measured excised head diameter, potentially exceeding the diameter of the implanted cup. An important counterpoint is the upper tail of the distribution, where a size difference exceeding 4 mm occurred in 18.17% of cases, approximately one in five patients. As it is not rare, it should not be interpreted as a contraindication to put a cup larger than the native head by more than 4 mm. If enforced rigidly, a 4‐mm cutoff could prompt frequent and potentially unwarranted wrong surgical decisions. A more clinically defensible approach is to treat a size difference > 4 mm as an intraoperative alert threshold that mandates a deliberate checkpoint. The surgeon should verify the femoral head measurement method (true equatorial axis and caliper placement), reassess reaming depth, version and hemispheric containment, and confirm that the selected cup size is not inadvertently compensating for unrecognized bone deficiency or eccentric reaming. Only after these elements are reconciled should a change in implant size or strategy be considered. The goal is not to simply stop further reaming and implant a smaller cup as a reflex response to the measurement. This is clinically relevant because an unexpected discrepancy at this stage may reflect not only oversizing, but also a non‐anatomic acetabular reconstruction. A study showed that even TAL‐guided or TAL‐based kinematic alignment of cup anteversion may not consistently satisfy functional requirements in THA, particularly when assessed against combined sagittal index or patient‐specific safe‐zone criteria [17]. Moreover, acetabular cup position has been linked to postoperative iliopsoas impingement after THA [25].

The intra‐operative measurement of the femoral head is an attractive technique because it provides a rapid, real‐time, cost‐free sizing reference and eliminates the risks of radiographic magnification and patient positioning, both of which can compromise the reliability of 2D templating [1, 9]. 2D templating generally demonstrates good accuracy in predicting component sizes [27], so it should be viewed as a probabilistic estimate rather than a fixed absolute. This limitation reflects the inherent constraints of standard preoperative planning with 2D radiographs, where radiographic magnification introduces scaling errors and measurements are sensitive to patient positioning [9]. At the higher end of the technological spectrum, CT‐based 3D planning in robotic‐assisted arthroplasty has demonstrated the capacity to achieve near‐perfect cup size prediction [12]. Nevertheless, CT‐based workflows add radiation exposure and cost, and their use is constrained by system availability and institutional resources. As such, intra‐operative femoral head measurement is best framed not as a replacement for preoperative planning, but as a vital, independent verification step. It serves as a real‐time intra‐operative check that can validate, or challenge, the planned size, particularly in settings when CT planning is inaccessible or when conventional preoperative templating remains uncertain. This added safeguard can help prevent gross sizing errors by flagging obvious outliers before final implantation.

This study has several limitations. The evidence base remains relatively small (four studies) and is largely observational, which limits the statistical reliability of heterogeneity estimates and reduces the precision of the PIs. Consequently, the proposed >4 mm alert figure should be interpreted as a hypothesis‐generating, descriptive benchmark rather than a validated clinical cutoff. However, the total number of hips included is substantial (631 hips), and the included studies used broadly comparable intra‐operative measurement approaches, using calipers, which supports pooling. Notably, this meta‐analysis quantifies a sizing relationship but does not report patient outcomes or complication rates. The *I* ^2^ statistic of 98% reflects genuine variation in true sizing discrepancies across study populations, driven by differences in surgical technique, implant design and patient anatomy; this magnitude of heterogeneity directly precludes the derivation of a precise, universally applicable numeric threshold from these data. Therefore, the most appropriate next step is prospective, multi‐centre validation with standardized measurement tools (calibrated calipers), a clear definition of whether cartilage or osteophytes are included in measurement, and explicit linkage between size‐difference categories, intra‐operative events and patient‐centred outcomes.

## CONCLUSION

The implanted cup averaged 2.93 mm larger than the excised femoral head, with most differences between 0 and 4 mm. Femoral‐head sizing should be used as a rapid, no‐cost intraoperative check that complements, not replaces, preoperative templating. A cup‐head mismatch threshold of >4 mm should prompt a deliberate pause to re‐verify caliper technique, reaming progression, containment and templating assumptions before deciding on component size.

## AUTHOR CONTRIBUTIONS

**Youssef Jamaleddine** and **Chahine Assi**: Conceptualization. **Ralph Maroun** and **Youssef Jamaleddine**: Writing—original draft. **All authors**: Writing—review and editing. **Youssef Jamaleddine** and **Chahine Assi**: Supervision. All authors read and approved the final manuscript.

## FUNDING INFORMATION

The authors have no funding to report.

## CONFLICT OF INTEREST STATEMENT

Pascal Kouyoumdjian has received consultant honoraria and speaker honoraria from Stryker and Lepine. The remaining authors declare no conflicts of interest.

## ETHICS STATEMENT

The authors have nothing to report.

## Supporting information


**Supplementary file**: PRISMA Chceklist.

Supporting File 1

## Data Availability

All data analysed in this study are derived from previously published studies cited in the reference list. Extracted data supporting the findings of this study are available from the corresponding author upon reasonable request.
