# Supplementary material for: Acetabular cup‐native femoral head size discrepancy in primary total hip arthroplasty: A meta‐analysis on intraoperative sizing verification
Source: J Exp Orthop. 2026 May 22;13(2):e70782. doi: 10.1002/jeo2.70782 (PMC13239272; doi:10.1002/jeo2.70782)
Supplement: Supplementary file 2 — Supporting File 1 [file JEO2-13-e70782-s001.pdf]

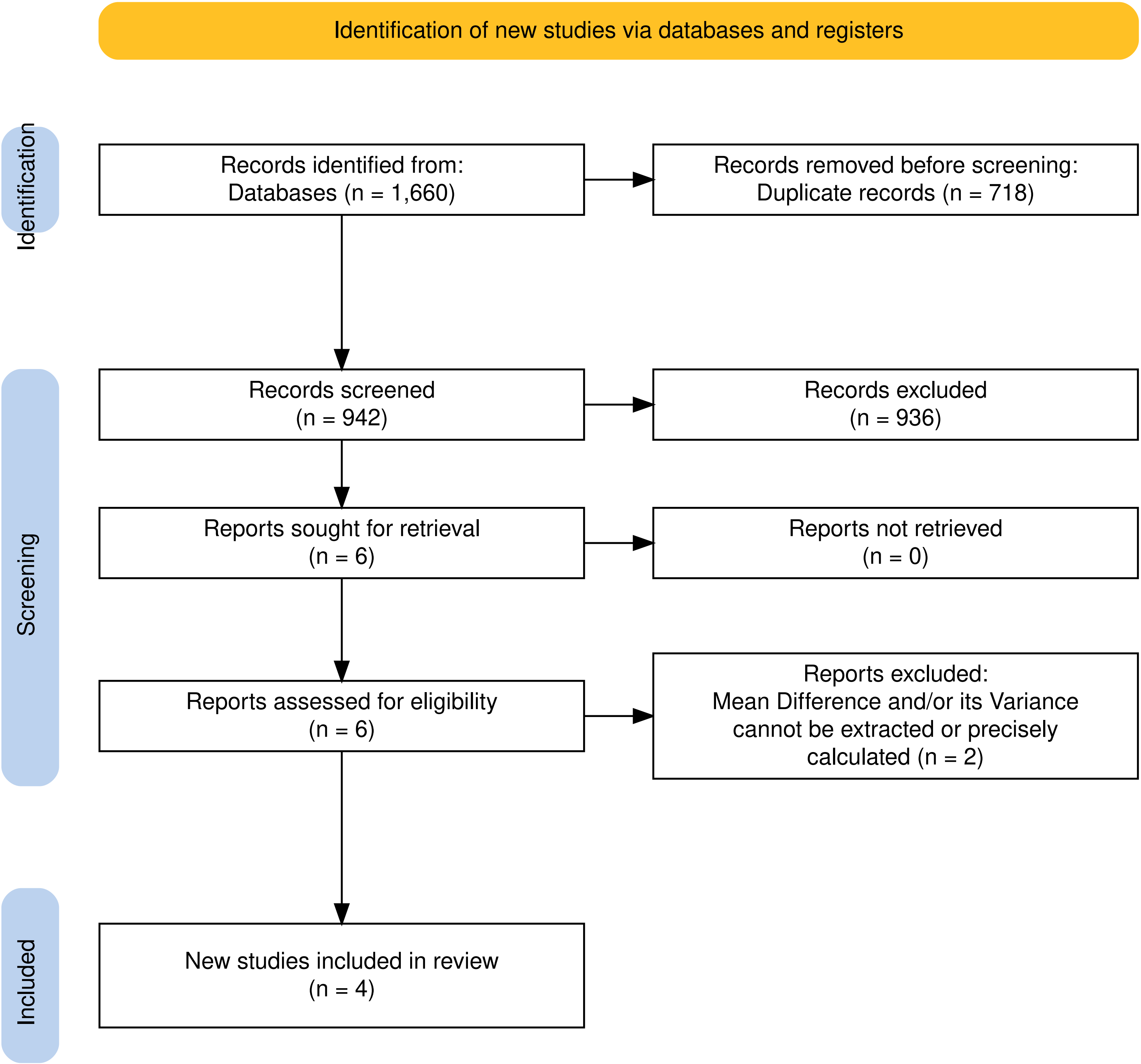
Identification of new studies via databases and registers
Identification
Records identified from:
Databases (n = 1,660)
Records removed before screening:
Duplicate records (n = 718)
Screening
Records screened
(n = 942)
Records excluded
(n = 936)
Reports sought for retrieval
(n = 6)
Reports not retrieved
(n = 0)
Reports assessed for eligibility
(n = 6)
Reports excluded:
Mean Difference and/or its Variance
cannot be extracted or precisely
calculated (n = 2)
Included
New studies included in review
(n = 4)
